# Towards Insect-Friendly Road Lighting—A Transdisciplinary Multi-Stakeholder Approach Involving Citizen Scientists

**DOI:** 10.3390/insects12121117

**Published:** 2021-12-14

**Authors:** Sibylle Schroer, Kat Austen, Nicola Moczek, Gregor Kalinkat, Andreas Jechow, Stefan Heller, Johanna Reinhard, Sophia Dehn, Charis I. Wuthenow, Martin Post-Stapelfeldt, Roy H. A. van Grunsven, Catherine Pérez Vega, Heike Schumacher, Leena Kaanaa, Birte Saathoff, Stephan Völker, Franz Hölker

**Affiliations:** 1 Leibniz Institute of Freshwater Ecology and Inland Fisheries, 12587 Berlin, Germany; kat.austen@igb-berlin.de (K.A.); gregor.kalinkat@igb-berlin.de (G.K.); andreas.jechow@igb-berlin.de (A.J.); stefan.heller@igb-berlin.de (S.H.); johanna.reinhard@igb-berlin.de (J.R.); catherine.perez@igb-berlin.de (C.P.V.); franz.hoelker@igb-berlin.de (F.H.); 2 PSY: PLAN Institute for Architectural and Environmental Psychology, 10245 Berlin, Germany; moczek@psyplan.de; 3 Museum für Naturkunde Berlin, Leibniz Institute for Evolution and Biodiversity Science, 10115 Berlin, Germany; 4 NABU RV Westhavelland e.V., Milower Land, 14715 Brandenburg, Germany; aube@nabu-westhavelland.de; 5 Umweltzentrum Fulda-Zentrum für Nachhaltigkeit, Gartenkultur und Tierpädagogik e.V., 36041 Fulda, Germany; Charis.Wuthenow@fulda.de; 6 Naturpark Nossentiner/Schwinzer Heide e.V., 19395 Plau am See, Germany; martinpost1986@gmail.com; 7 Dutch Butterfly Conservation, P.O. Box 506, 6700 AM Wageningen, The Netherlands; roy.vangrunsven@vlinderstichting.nl; 8 Department of Biology, Chemistry, and Pharmacy, Institute of Biology, Freie Universität Berlin, 14195 Berlin, Germany; 9 Chair of Lighting Technology TU Berlin, 10587 Berlin, Germany; heike.schumacher@tu-berlin.de (H.S.); leena.kaanaa@tu-berlin.de (L.K.); birte.saathoff@tu-berlin.de (B.S.); stephan.voelker@tu-berlin.de (S.V.)

**Keywords:** Ephemeroptera, Trichoptera, species conservation, light pollution, artificial light at night, biodiversity, darksky, insect decline, insect diversity, nightscapes, street lighting, obstructive light

## Abstract

**Simple Summary:**

Road lighting is a service provided at night, mainly to ensure the secure and safe passage of humans. However, lighting at night can have adverse effects on insects or ecosystems, which are not yet considered in planning. Here, we introduce a comprehensive approach for the design and implementation of a novel insect-friendly road luminaire. The lighting design provides an optimized radiation geometry that avoids emissions at the trajectory height of insects, reduces the attraction of insects and the fragmentation of their habitats, and at the same time provides adequate night-time illumination in residential areas. The effects of the new design on insect behavior and night sky brightness will be evaluated two years before and two years after the change of the road luminaires and additionally in a direct comparison, as some luminaires of the old design will remain as controls. Citizen scientists are involved in the identification of insects and the measurement of night sky brightness. A broad public engagement program also highlights discussions about the competing interests of different stakeholders in lighting design, explicitly including the effects of illumination on insect fauna and biodiversity.

**Abstract:**

(1) The project “Tatort Streetlight” implements an insect-friendly road light design in a four year before–after, control–impact (BACI) approach involving citizen scientists. It will broaden the stakeholder interests from solely anthropogenic perspectives to include the welfare of insects and ecosystems. Motivated by the detrimental impacts of road lighting systems on insects, the project aims to find solutions to reduce the insect attraction and habitat fragmentation resulting from roadway illumination. (2) The citizen science approach invites stakeholders to take part and join forces for the development of a sustainable and environmentally friendly road lighting solution. Here, we describe the project strategy, stakeholder participation and motivation, and how the effects of the alternative road luminaire and lighting design can be evaluated. (3) The study compares the changes in (a) insect behavior, (b) night sky brightness, and (c) stakeholder participation and awareness. For this purpose, different experimental areas and stakeholders in four communities in Germany are identified. (4) The project transfers knowledge of adverse effects of improperly managed road illumination and interacts with various stakeholders to develop a new road lighting system that will consider the well-being of street users, local residents, and insects.

## 1. Introduction

Road lighting is installed by humans to meet human needs. However, it can have unintended effects on the well-being of insects and ecosystems and the services they provide to humans. “Tatort Streetlight”, named in honor of the German crime series “Tatort”, meaning “crime scene”, is a project that observes and investigates the night-time crimes perpetrated against nocturnal flying insects on illuminated streets. This study takes a new perspective on the design of road lighting systems via the involvement of diverse stakeholders. The approach taken involves transdisciplinary citizen science, education, and awareness and invites stakeholders to contribute to a better understanding of an improved luminaire and lighting design for both human benefit and insect welfare.

Artificial light at night (ALAN) affects flora and fauna [1,2,3] and can have an impact on human well-being (e.g., [4,5]). The adverse effects of ALAN on the environment can include whole ecosystems, cascading effects and even threatening biodiversity [6,7]. Freshwater ecosystems are important sources of biodiversity but the impacts of ALAN on them are not well studied [8]. The few studies available indicate strong effects on freshwater biodiversity [9,10,11]. Furthermore, improperly managed, excessive, and unnecessary ALAN degrades the night sky, an important cultural and aesthetic resource [12,13,14], and causes massive energy consumption [15,16]. ALAN is growing exponentially in radiance and extent with a global average increase of more than 2% per year observed with satellite data [17]. Within the “Tatort Streetlight” project, we want to test the effects of road lighting on the trajectory of insects close to freshwater bodies, develop solutions against the adverse effects of roadway illumination on insects, and increase awareness of the adverse effects of ALAN on the environment.

A dramatic insect decline has been reported in Germany by Hallmann et al. (2017), among others, with a 75% reduction of insect biomass between 1989 and 2013 [18]. The decreasing number of insects could result in a rapid reduction of ecosystem services such as natural pest control, pollination, conservation of soil structure and fertility, nutrient cycling, and biodiversity maintenance (e.g., [19]). This decrease is partly explained by changes in land use and increased use of pesticides. However, conclusive explanations are still lacking, especially for the decline in protected nature areas. The strong decline in nocturnal insect species is particularly striking [20]. This insect group makes up about half of all insect species described worldwide [6]. It was shown that the areas studied by Hallmann et al. (2017) [18] were predominantly in landscapes with a moderate to high level of light pollution, suggesting a connection between light pollution and insect mortality [21]. Furthermore, van Grunsven et al. (2020) found that ALAN can have a negative effect on moth populations in the long term [22]. Streetlights can have a so-called “vacuum cleaner effect”, attracting insects from adjacent habitats [1] (pp. 281–304), which can create barrier effects along illuminated streets and cause fragmentation of nightscapes and a limitation of insect dispersal [23].

Some of the best long-term insect monitoring data to date have been provided by citizen scientists [24]. Insect diversity and its function in ecosystems has become an important research field for citizen science [25,26,27]. Citizen scientists were crucial to both the study by Grunsven et al. (2020) [22], for which citizen scientists counted the moths at most locations, and the aforementioned study by Hallmann et al. (2017) [18], which is the work of an organization of professional entomologists together with citizen scientists, who published the analyses of decreasing biomass trends derived from long-term insect monitoring data. As such, citizen science approaches have already been adopted at the political level [28]. Citizen science has also become an indispensable research tool to track the brightening of the night sky at a global scale [29,30]. Citizen scientists are using hand-held mobile or permanently installed radiometers, questionnaires, or apps to determine ALAN sources or skyglow [31].

The “Tatort Streetlight” project builds on these traditions of citizen science approaches in ALAN and insect diversity research, which have until now not been connected, to amplify awareness and environmental education, as well as providing a huge scientific data set. The project promotes the mutual exchange of relevant topics and obstacles, highlights the issues and social challenges, and acts as a bridge between research and applied knowledge [32]. The involvement of citizen scientists is well established, and they not only contribute data but also help to implement effective conservation measures [33].

This article presents a project strategy proposal for the citizen science approach, introduces the different conditions in the four project areas, and summarizes the preliminary results of the first project year. The results describe the starting point that can be used to move the new, insect-friendly lighting design into municipal practice and to expand awareness of it among all stakeholders. To our knowledge, this is the first approach to communicate between stakeholders with ecological and lighting planning interests for the urgently needed development of an insect-friendly road luminaire and lighting design. We present the technical implementation of an insect-friendly luminaire, taking into account the lighting parameters for road safety. We hypothesize that the new road lighting design and the stakeholder engagement will be able to (a) decrease insect attraction to single road luminaires and the fragmentation of the habitat, (b) reduce the overall night sky brightness, and (c) increase the awareness of all stakeholders.

## 2. Materials and Methods

Within the “Tatort Streetlight” project we develop a new luminaire and lighting design with optimized radiation geometry for roadways in order to reduce the impacts of emissions on insect trajectories, with a focus on aquatic insects. The “Tatort Streetlight” project is following the recommendations of Kalinkat et al. (2021) for ALAN research on insects to provide the following: (a) independent samplings and control treatments in different partner communities with similar roadway lighting at freshwater bodies; (b) adequate resolution of insect sampling and analysis at the luminaires, from the water and in adjacent habitats; (c) measurement of ALAN and its multiple dimensions [34]. A 4 years BACI (before–after, control–impact) approach is initiated to allow a comparison of the before and after conditions in two consecutive years, before and after the transition. The direct comparison of the old and the new road lighting designs serves as the control, especially if the environmental conditions after the transition to the new lighting design differ, for example due to weather or insect occurrence. Secondly, stakeholders such as residents and authorities will have the opportunity to judge the different road lighting designs in a direct comparison.

Stakeholders with diverse backgrounds are identified and invited to evaluate the optimization of insect-friendly road lighting. These are defined as anyone with an interest in the project or who could be affected by the research and is directly or indirectly involved. Various risk aspects of light pollution are addressed, such as reduced light emission in non-target areas, impacts on insect diversity, as well as safety and security. The citizen science approach serves as a tool for knowledge transfer in terms of communication to stakeholders and for expanding public awareness [35].

### 2.1. Identification of Partner Communities

All project areas either already have the status of star park or intend to become one. Star parks are areas that implement strategies for the protection of a naturally dark sky. The communities commit to rules for night-time outdoor illumination prescribed by the International Dark Sky Association (IDA), which has so far certified over 100 areas globally within the international dark sky places program (https://www.darksky.org/our-work/conservation/idsp/, accessed on 9 December 2021). Further, the project areas can provide centers for environmental education or community halls in order to offer workshops and events to the public. The respective partner communities commit to the four-year experimental period, in which the existing road lighting design is subject to changes. These changes envision a new lighting layout over a length of at least 100 m close to a freshwater body. Parts of the existing road lighting installation will be left in the old status to be able to compare the effects directly.

The search for partner communities was initiated at the Second Star Park Initiatives Workshop in Germany, 2018. Many participating astronomers were interested in seeing their communities being involved in this project. However, comparable sites at freshwater bodies needed to be identified, the authorities needed to be convinced, and the road lighting design needed to be in a rather comparable way between the project areas.

Four communities finally provided these conditions, namely the cities Krakow am See (in the federal state of Mecklenburg-Vorpommern) and Fulda (Hessen) and the communities Neuglobsow in Stechlin-Ruppiner Land and Gülpe in Westhavelland (both Brandenburg). The Westhavelland Nature Park received the star park status in 2014, and the city of Fulda was the first city in Europe to receive certification as a star city in 2019. The communities of Krakow am See in the Nossentiner/Schwinzer Heide Nature Park and Neuglobsow in the Stechlin-Ruppiner Land Nature Park are currently planning the application for the certification as a star park.

### 2.2. Technological Development of an Insect-Friendly Road Lighting Design

The aspects of the lighting design were defined by lighting engineers at the Technical University of Berlin (TU Berlin) in cooperation with ecologists and physicists at the Leibniz Institute of Freshwater Ecology and Inland Fisheries (IGB). As the lighting design will be implemented within inhabited areas, it was necessary to take into account current guidelines for security and safety. Although the technical standard EN 13201 does not consider ecological aspects, many authorities recommend following EN 13201 in the absence of alternatives. Thus, the new road lighting should follow the EN 13201-2 lighting standard (Performance Requirements for Road Lighting). The standard specifies different lighting classes for motorized traffic (M-classes) and for pedestrians (P-classes) with corresponding requirements for the illuminance (P-classes), luminance (M-classes), uniformity, glare, and surround ratio [36].

The areas that qualified for the studies in this project are mainly pedestrian and bicycle paths or roads with speed below 30 km/h or passage restrictions. Therefore, P-classes (classes for mainly pedestrian and low-speed traffic) instead of M-classes (for mainly motorists and faster road users) were used for this study. Lighting classes are further specified by the parameters traffic speed, volume and composition, parked vehicles, and ambient luminance [37]. The criteria for lighting class P4 were the target of the new luminaire design due to the focus on roads with restricted motorized traffic in the project areas. In order to maintain uniformity, the actual values must not exceed 1.5 times the intended values for the lighting class. The illuminance values (*Ē*_av_—average horizontal illuminance; *E*_min_—minimum horizontal illuminance; *E*_av,max_—maximum average horizontal illuminance) of class P4 and the next higher and lower classes are indicated in Table 1.

**Figure 1:**
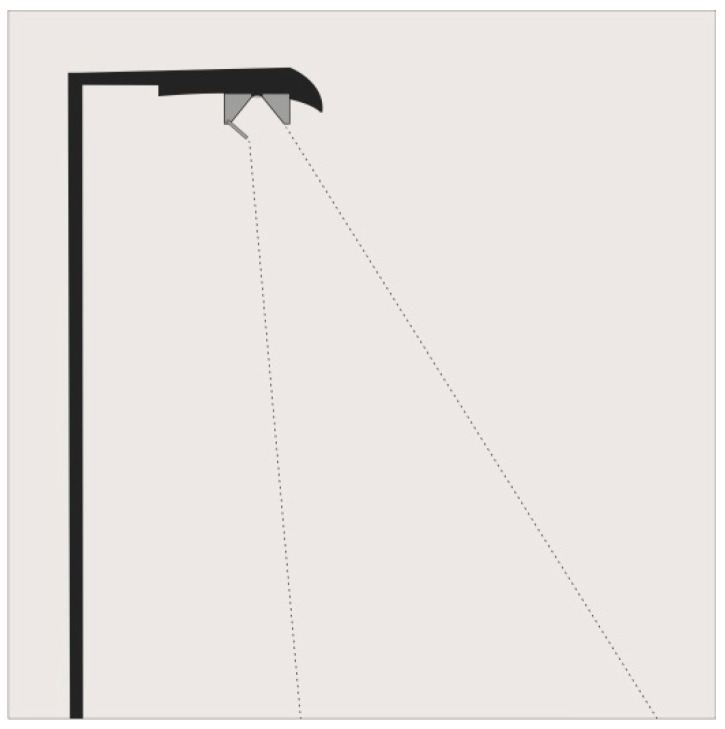
Design of an insect-friendly road luminaire. Mast arm is matt black to reduce reflection. The light point overhang from the center of the luminous area is 1.0 m without any slope. The road lighting design aims at a strict light distribution curve with a luminous intensity of 0 cd in the non-target area. Illustrated by Pérez-Vega.

The approach of our study is to develop light distribution curves that direct the emission of light only to where it is needed with a wide angle at the utilization area and shielded light emission at the light source using shutters (Figure 1). The aim is that the light points will not be visible from adjacent water bodies, floodplains, or other habitats.

The current luminaires in the project areas are mostly common decorative post-top mounted luminaires installed in public amenity areas such as parks. The cylinders are made of glass or plastic and are bright, with some degree of glare. These old lighting systems usually do not contain an optical element inside the luminaires.

The road geometries of the project areas, i.e., road widths as well as pole heights and pole distances, are shown in Table 2. Pole height/distance ratios above 5 can cause either high glare, characterized through high threshold increment (TI-value), or bad uniformity, which can result in low traffic safety. When retaining a relatively low luminaire mounting height and a steep angle of incidence of light, in order to reduce light emission in the insects’ flying habitat [38], it is recommended to use smaller pole distances to achieve sufficient safety and security. Thus, communities face the fundamental decision of either investing in a larger number of poles or accepting reduced illuminance or even dark zones between the luminaires.

### 2.3. Experimental Design

The so-called “vacuum cleaner effect” is a metaphor for the high attraction of various insect orders to road lights, resulting in the removal of their local living community [1] (pp. 281–304). Aquatic insects, such as caddisflies and mayflies (Ephemeroptera and Trichoptera), are attracted from distances of more than 40 m [39]. Degen et al. (2016) measured an attraction radius for moths of over 20 m when exposed to high-pressure sodium lamps (HPS) at night [23]. Artificial lighting systems mounted in rows with distances between each luminaire of 45 m and less have been shown to create a “barrier effect” that disrupts insects’ flight patterns. This means that insects, while on trajectory flights between two poles of 45 m distance or less, will be attracted to either one or the other point source of light, rather than entering the habitat behind the lighting system. Barrier effects by road luminaires are also well described for bats. The width and the intensity of the illuminated area correlate with the success of bats in crossing the pathway [40].

The aim of the new road luminaire and lighting design is to reduce both the attraction to single light points and the barrier effect. In a BACI approach, the behavior of various insect orders and suborders will be measured before and after the conversion to the new road lighting design.

#### 2.3.1. Monitoring Design at the Experimental Field Site

**Figure 2:**
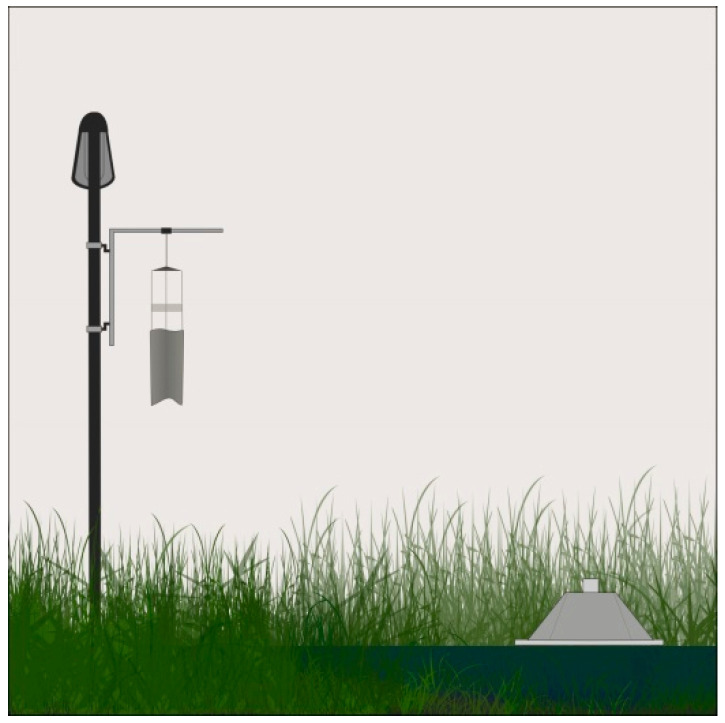
Insect flight trap (left) and emergence trap in the water (right) used to estimate the occurrence of flying insects in the trajectory at the luminaire and emerging insects from the water body, respectively. Illustration by Pérez-Vega.

The first installation of the new road lighting design will be conducted at an established experimental field site approximately 20 km east of Gülpe, one of the participating communities. The field site was specifically designed to measure the barrier effects of road lights on insects for the “Loss of the Night” project [41]. At this field site, the effects of artificial lighting on a formerly ALAN naïve ecosystem have been studied since 2012 (see [9,23,42]). Insect emergence from the water body is measured using emergence traps, while the occurrence of flying insects at the luminaires is measured using air eclector traps (Figure 2).

In order to measure the insect behavior under comparable natural lighting conditions, the established protocol includes sampling once per month at the time of the vanishing half-moon. Air eclector traps are activated for a total of 24 h, whereby the night-time from dusk till dawn is sampled separately from the daytime from dawn until dusk, which allows temporal standardization of trapping periods. The emergence traps are treated the same. Additionally they are activated for a whole week, which allows a better resolution of insect emergence.

At the field site, 12 luminaires are installed in three rows. The light sources currently installed in the field are Teceo LEDs with a correlated color temperature of warm white equivalent to 3000 Kelvin from Schreder. The new road lighting design will be installed after the winter season in 2021. Previous studies have shown that this common road lighting design attracts higher rates of insects to the luminaires located at the corners and edges of the field site when compared to the luminaires positioned in the middle, indicating a barrier effect [23]. In accordance with the studies by Degen et al. (2016) [23] and Manfrin et al. (2017, 2018) [9,42], we hypothesize that the improved light distribution geometry will attract fewer insects and that equal numbers will be attracted to the single luminaires.

#### 2.3.2. Monitoring Design at the Selected Communities

**Figure 3:**
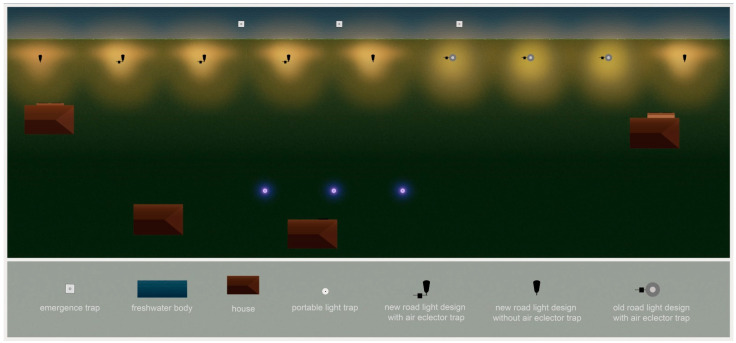
Experimental design for use in the partner communities. From a row of luminaires close to a water body six, luminaires are equipped with insect flight traps. Three luminaires will be kept in the old design for a 2 years direct comparison. Emergence traps in the water are used to estimate the insect emergence from the water body and light traps are used to test the potential of the trajectory through the row of road luminaires. Illustration by Pérez-Vega.

After the primary test phase in late autumn 2022, the novel, insect-friendly road lighting design will be installed at the partner communities for the four year BACI insect monitoring. Similar to the experimental field site, insect air eclector traps are mounted at each luminaire and three emergence traps are implemented in the closest freshwater body. (A map of the location of the partner communities and the detailed position of the luminaires, which are involved in the studies can be found in the Supplementary Materials, Figures S1–S5). The luminaire distribution in the community locations does not recreate the experimental set-up in multiple rows of the experimental field site. Therefore, additional light traps are used to measure the barrier effect, which are positioned in resident gardens or parks opposite the lights near the water bodies at distances of at least 20 m (Figure 3). These portable light traps are based on Heath traps (Heath, 1965) and consist of a light source with three acrylic vanes in a funnel that rests in a 27 L container with egg cartons inserted in it. As a light source, we use a 50-cm-long 2835 SMD-LED-strip with peak emissions at a wavelength of about 400 nm wrapped around a 21 cm PVC tube. The LED strip is powered by a power bank and switched on via a light sensor. These traps are slightly more effective in catching moths than portable Heath traps with a 6 W actinic lamp and likely have a comparable attraction radius [43]. We hypothesize that after the transition to the new light design more insects will be caught within these mobile light traps, due to reduced attraction to the single road luminaires. The comparison will be analyzed according to the method of Degen et al. (2016) [23].

Both at the field site and at the project communities, the monitoring will be conducted at the vanishing half-moon every four weeks, depending on weather conditions +/−3 days. This adds up to eight trial days in the season from March until October.

### 2.4. Measuring the Effects on the Night Sky Brightness and Night-Time Radiance

The environmental impact of ALAN is manifold. Adverse effects on wildlife and habitat have been described by both direct light emission and brightening of nightscapes by indirect light—the so-called skyglow [44]. The sum of all ALAN, including road illumination, private lighting, and other lighting, contributes to both horizontal and upward light emission. These ALAN emissions are scattered within the atmosphere and diverted back to the Earth’s surface, observable as skyglow that results in increased night sky brightness. The natural night sky brightness of a moonless night in an area without light pollution is on the order of 0.17–0.25 mcd/m^2^, with an illuminance of the order of 1 mlx. However, urban skyglow can be several thousand times brighter than that of the natural night sky brightness [45] and illuminance levels are of the order of 1 lx [46,47], which is much brighter than peak full-moon (<0.3 lx) [48].

To estimate the upward ALAN light emission in the experimental areas, night-time satellite data from the visible and infrared radiometer suite (VIIRS) day–night band (DNB) are used. The webapp “radiance light trends” [49] uses VIIRS DNB monthly composites, which provide the night-time radiance in the spectral band between 500 nm and 900 nm for (nearly) all months between 2012 and 2020 [50] (https://lighttrends.lightpollutionmap.info, accessed on 9 December 2021). In high-latitude regions, the summer month data are not provided because of the lack of astronomical night and resulting solar straylight. With this webapp, the upward night-time radiance can also be monitored using a BACI approach before, during, and after the experiment, as well as monitoring the trend of radiance (increase or decrease in brightness) during the experiment and before. The resolution of the VIIRS DNB satellite data is 740 m (selectable pixel in the app might be smaller). The region of interest as a single pixel will be centered in the different experimental areas and charts of radiance measurements are recorded.

The upward radiance can be linked to skyglow and the night sky brightness by modeling [31], but it is difficult to include the dynamics of skyglow due to weather (clouds, snow, etc.) [51] and light usage in these models [52,53]. Thus, it is well established to monitor the night sky brightness at the zenith from the ground with small radiometers (with only near-photometric sensitivity), such as the “TESS” stars4all radiometer (Fundacion stars4all, Madrid, Spain) or the Sky Quality Meter (SQM, Unihedron, Grimsby, Canada) [54]. Alternatively, digital cameras can provide more comprehensive information about the night sky brightness [55].

The TESS radiometer is a rather new device for citizen scientists. It can be connected to a Wi-Fi network, then the data are uploaded to a globally growing night-time sensor network (https://tess.stars4all.eu/, accessed on 9 December 2021). Since 2006, The “Globe at Night” webapp has allowed citizen scientists to upload measurements via computers or smartphones (https://www.globeatnight.org/webapp/, accessed on 9 December 2021). The app invites citizen scientists to indicate the visibility of single stars within the Orion star constellation. Since 2012, the “Loss of the Night” mobile app has allowed citizen scientists to indicate the visibility levels of different stars (https://actionproject.eu/citizen-science-pilots/loss-of-the-night/, accessed on 9 December 2021). The app guides users across the sky to certain stars. On the basis of the magnitude of the observed stars, the app calculates the local night sky brightness. The data can be sent to the global “Globe at Night” database. All of these citizen science apps and uploaded SQM data can be viewed at the My Sky at Night website (http://www.myskyatnight.com, accessed on 9 December 2021).

In “Tatort Streetlight”, at least one radiometer will be installed near each of the project locations. Residents will be encouraged to either install a TESS radiometer or use an SQM in their gardens for long term monitoring or to use handheld versions of these devices for mobile measurements. Citizen scientist groups will be motivated to regularly determine the naked eye limiting magnitude closely related to the night sky brightness [29] using the “Globe at Night” or the “Loss of the Night” mobile app.

### 2.5. Communication, Environmental Education, Participation, and Citizen Science Approach

In order to plan a successful communication strategy, it is first important to know people’s knowledge, attitudes, and experience of the subject in question [56]. We hypothesize the public knowledge about adverse ALAN impacts on health, well-being, and the environment to be rather low but growing due to the increasing information about light pollution in the media. All communication strategies in our project will first arouse curiosity, provide substantial information on the different aspects, and then raise awareness with regard to necessary changes. It is important to take possible points of criticism into account as early as possible. For example, it could turn out to be an obstacle that night-time darkness is associated with the fear of a lack of security and safety. Furthermore, unforeseen ecological incidences could coincide with the project’s actions and may be associated with the changed lighting situation, e.g., the appearance of excessive mosquito biomass due to favorable weather conditions. Furthermore, the knowledge and importance of nocturnal insects and the motivation to volunteer in the project might be rather low because of the generally poor image of insects.

The project has and will continue to reach out to the local public in the partner communities and make use of a targeted social media strategy to open the research to participation from diverse groups.

Workshops will be offered to draw the interest of the local public to nocturnal wildlife and ecological systems, insect occurrence, insect behavior, and their functions, and also on outdoor illumination and the impacts on the environment. The first target of the workshops is to increase the interest of the public in the local insect fauna and to gain human resources for the research field entomology. The second target of the workshops is to increase the awareness about the adverse effects of ALAN on human well-being and the environment. The public understanding of this issue is crucial to avoid the risk of residents compensating for the lower public luminance in experimental areas by installing more private lighting, thereby causing rebound effects in the experimental zone [57].

The workshops’ target groups are inclusive, representing important stakeholders from the whole of the society (see a–f). The workshops will be tailored to the needs of each target group in terms of size, timing, and location, as follows:

- Small children in the kindergarten, who are often a “door opener” to draw the interest of grandparents and elderly people;
- School pupils of middle age classes, grades 7 to 10, who offer the highest potential to reach out to diverse communities;
- Teachers and education staff, who make the contents of the workshops sustainable;
- Authorities for light and urban planning;
- Manufacturers of outdoor lighting products;
- The “Tatort Streetlight” target group, involving the citizen scientists.

Citizen scientists are not only reliable data providers. In our project, their task is crucial in identifying collected insects at the family and species level, as many citizen science experts can assist the project via their unique knowledge of certain insect groups [58]. The project also aims to connect the knowledge of isolated hobby entomologists. A platform for networking might help to discover more details about the impact of ALAN on certain insect taxa, e.g., single species or ecological function groups. Citizen scientists can assist workshops, help in identification in community halls, or borrow parts of the collection to identify insects in their private homes or at other institutes.

### 2.6. Socioeconomic Evaluation Strategy

The project “Tatort Streetlight” addresses different target groups, for each of which a separate evaluation will be developed. The mixed-media approach, with a combination of different techniques and instruments, follows the studies conducted by Coogan et al. (2020), Lyytimaeki and Rinne (2013), Moczek (2019), and Silver and Hickey (2020) [59,60,61,62]:

- For the citizen science part of the project, i.e., the insect monitoring, it is planned to use an online survey to assess problem awareness and attitudes towards the insect population and causes of decline, motivations to participate in the project, and the behavior of the participants. The surveys are conducted at the beginning of the volunteer engagement and will be repeated at the end of the funding period;
- Residents of the project communities are involved and interviewed twice in the form of group discussions. The aspects of interest will be their experiences and assessments of night-time light and current road lighting and the impacts of light on their own health and well-being and on insect diversity. The second study will take place after the new road lighting design has been developed and built up locally;
- Environmental education participants, especially students, are interviewed via online questionnaires a few days after attending the workshops;
- In addition, face-to-face interviews are conducted with various stakeholders, such as local businesses, tourism representatives, city staff, and others.

### 2.7. Data Management

Data collection follows the recommendations of de Sherbinin et al. (2021) [63]. All data and metadata generated in this project will be uploaded and indexed in an open data portal. This includes: measurements of night-time brightness; dates and locations of trap collection (including trap number and name of the collecting person); time and location of the identification; name of the identifying person and email contact or contact of the associated group; name of the supervising coordinator; taxonomic identification to at least the order level; images of insects if available. The data will be protected and identifiers treated anonymously according to the citizen science project guide (https://doi.naturkundemuseum.berlin/data/10.7479/c3y1-fw50, accessed on 9 December 2021). The datasets are accessible and published under cc-by 4.0 license. To make data entering and access available for all citizen scientists, the “Epicollect” platform is used (https://five.epicollect.net/project/tatort-streetlight, accessed on 9 December 2021). At this website, users have to register and data are treated anonymously, presenting only a given user name. From this platform, the data are verified and copied into a database hosted by the IGB.

## 3. Results

Here we describe in detail the developed study design, the mapping of stakeholders and communities, as well as the first preliminary monitoring results from the four communities. The project developments are organized into the following subchapters: (1) scientific insect monitoring results; (2) basis of the light emission measurements; (3) the first steps in terms of communication, environmental education, participation, and the citizen science approach.

### 3.1. Insect Monitoring

**Figure 4:**
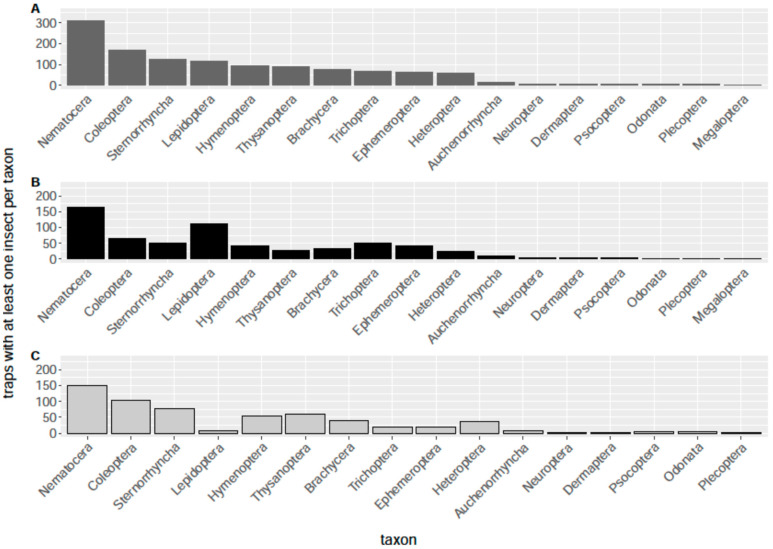
Distribution of taxonomic groups of flying insects in the air eclector and emergence traps, shown as the sum of all traps (**A**) for night (**B**) and day (**C**) only, respectively. The bars present the numbers of traps that had ≥1 of individuals of the specific groups to normalize peaks of occurrence of specific groups with very high individual sampling events (particularly Nematocera).

The first monitoring season in the partner communities was conducted with eight samplings from March until October 2020. Miscommunication between the lighting authorities and project coordination led to the half-night service problem, whereby some of the public lighting was not being turned off universally. For energy saving reasons, after 22:30 every second luminaire was turned off in the Krakow am See and Neuglobsow communities and all luminaires were turned off in Fulda. Thus, the first results for this monitoring period do not present the full spectrum of nocturnal insect species occurring throughout the night, except for the Gülpe project area. Despite these inconsistencies in our first monitoring season, we present a general and preliminary overview of broad taxonomic groups (mostly insect orders and suborders) and the respective numbers per trap and location that we sampled. Figure 4 provides an overview of the number of traps that were counted with at least one individuum per taxon, summarizing all eight sampling events during 2020.

**Figure 5:**
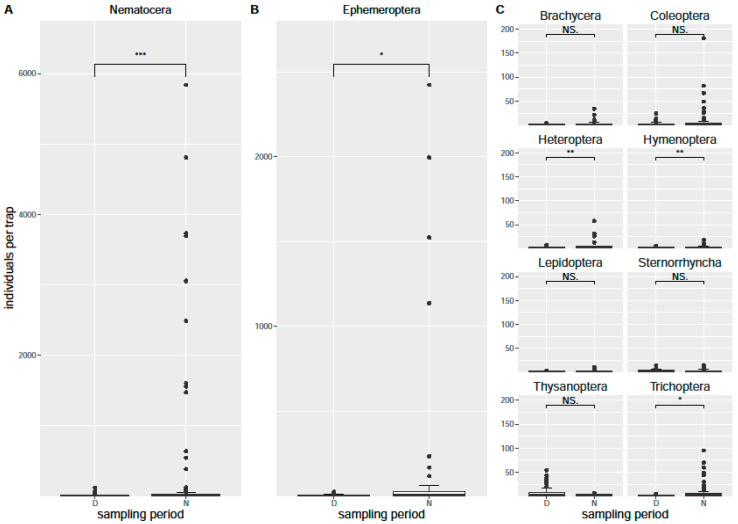
Pairwise comparison of night-time and daytime samples of flying insect orders and suborders in the air eclector traps of the four partner communities. Significant differences (*p* > 0.05) with higher numbers during the night-time were found for Nematocera (*p* = 2.5 × 10^−6^) (**A**), Ephemeroptera (*p* = 0.021) (**B**), Heteroptera (*p* = 0.0029), Hymenoptera (*p* = 0.0011), and Trichoptera (*p* = 0.025), all in panel (**C**).

The sampling results were temperature-dependent, especially the sampling dates in May and July, which were relatively cold and presented low numbers of insects (see Figures S6 and S7 in the Supplementary Materials). Samplings were conducted with no or only low rainfall. The predominant orders in the air eclector traps were Diptera (mainly the suborder Nematocera), Ephemeroptera, Coleoptera, Hemiptera (mainly the suborder Sternorrhyncha), Lepidoptera, Thysanoptera, Hymenoptera, and Trichoptera (Figure 4). On a single night and in a single air eclector trap, up to 800 insects were collected per hour. In the month of June, more than 2000 Nematocera were collected per hour in the sum of all air eclector traps in Gülpe and more than 700 Ephemeroptera per hour in Krakow am See (see Supplementary Materials, Figures S6 and S8). Subtracting Ephemeroptera and Nematocera due to their mass occurrence, still over 50 and 200 insects were collected per hour in Gülpe in the warmer months June and August, respectively (see Supplementary Materials, Figure S7). The comparison of the day and night-time occurrence rates in the eclector traps seemed to indicate higher activity at night-time for the orders Lepidoptera, Ephemeroptera, and Trichoptera (Figure 4). However, a statistical analysis (Wilcoxon rank sum test) revealed that the suborder Nematocera and the orders Ephemeroptera, Heteroptera, Hymenoptera, and Trichoptera were sampled significantly more often during night-time, while Thysanoptera was sampled significantly more often during daytime (Figure 5).

**Figure 6:**
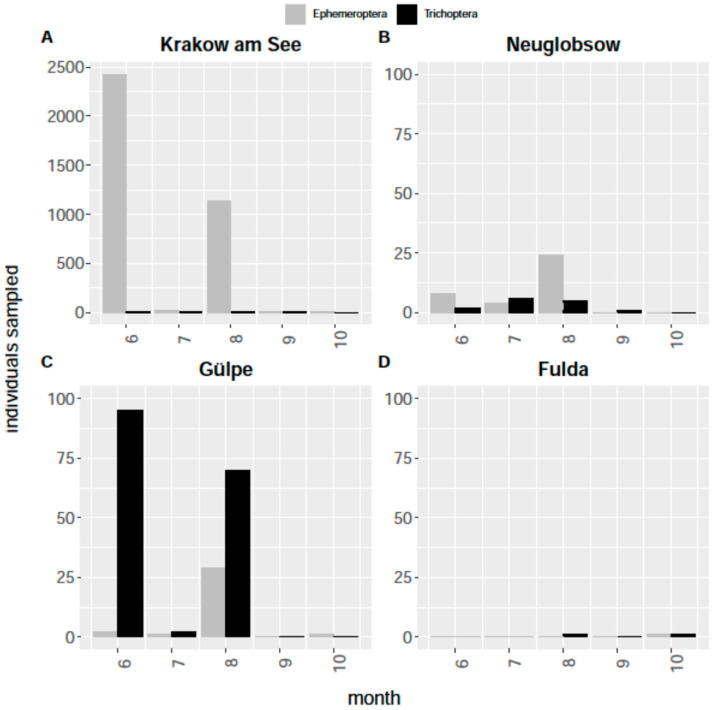
Total numbers for insect orders Trichoptera and Ephemeroptera trapped in the four partner communities from air eclector traps in the months June to October (6–10): (**A**) Krakow am See; (**B**) Neuglobsow; (**C**) Gülpe; (**D**) Fulda. Two mass emergence events of Ephemeroptera in Krakow am See resulted in high numbers (be aware of differences in *y*-axis scales).

In Figure 6, the occurrence rates of the orders Ephemeroptera and Trichoptera are explicitly presented for all sampling locations. These orders are 100% aquatic and can be expected to have traveled from a nearby freshwater body.

The results of the emergence trap collections mirrored the collections from the air eclector traps, indicating that indeed a high ratio of the collected insects emerged from the freshwater body. However, the high peaks of Nematocera and Ephemeroptera were not observed in the emergence traps. The main insect order in the emergence traps was again Diptera, mainly of the suborder Nematocera and to a lesser amount of the suborder Brachycera. Other orders regularly found in the emergence traps were Ephemeroptera, Coleoptera, and Trichoptera. (Detailed maps of the locations of the traps and more detailed figures presenting the monitoring results can be found in the Supplementary Materials).

### 3.2. Measurements of Night Sky Brightness and Night-Time Radiance

The experimental areas have relatively low night-time radiance levels comparable to the experimental field site. The towns Krakow am See and Fulda have the highest night-time radiance, with Fulda having 2 to 12 nW/cm^2^sr and Krakow am See having 0.5 to 2 nW/cm^2^sr. The two rural areas, Neuglobsow and Gülpe, have lower radiances of the order of 0.1 to 1 nW/cm^2^sr. The public lighting conditions have not changed in any of the study areas since 2012. In the study areas in Krakow am See, Gülpe, and Fulda, high-pressure sodium lamps are installed, while in Neuglobsow mercury vapor lamps are used.

### 3.3. First Steps in Communication, Environmental Education, Participation, and the Citizen Science Approach

Here we present the first communication steps and strategies undertaken in the partner communities in 2020. We first describe the conditions and associated strategies of the four partner communities and later the obstacles we experienced in communication with the public due to the special COVID-19 pandemic situation at the start of the project.

Citizen scientists have been studying bats in Krakow am See since 1983. In cooperation with this long-term citizen science project on bats, “Tatort Streetlight” provides information on insects for an already established bat trail and in workshops with a focus of young children as multipliers.

In Neuglobsow, workshops were designed to initially introduce pupils and their teachers to the subject of light pollution, up-to-date scientific results, and the effects on insect biodiversity. Upon introduction by an expert, participants were asked to sort given samples of insects captured during the season at the taxonomic order or suborder level. The students helped to set up the traps for the monthly monitoring and they were introduced to the local ecosystem and the importance of insects in aquatic ecosystems at the nearby lake. Furthermore, an awareness workshop was conducted to initiate the cooperation with “Jugend Hackt” at the “Verstehbahnhof” in Fürstenberg. “Jugend Hackt” is an organization that provides locations and materials to establish digital networks, while the “Verstehbanhof” is one of the “Jugend Hackt” locations, which offers teenagers opportunities to develop sensor networks to monitor environmental pollution. During a day-long workshop, “Tatort Streetlight” took the students of “Verstehbahnhof” on a light pollution excursion. A “TESS” radiometer was installed on the rooftop and the participants—students between 15 and 18 years—were asked to develop future lighting in the form of a citizen science co-design task (http://doi.org/10.5281/zenodo.4963835, accessed on 9 December 2021).

In Gülpe, the participation and motivation of local residents is organized through environmental actions from NABU, a German environmental NGO. The area is located in the Havel floodplain, where entomological experts have been observing the insect fauna since the 1980s. NABU was formed on this basis of establishing hobby entomologist expert networks around the “Tatort Streetlight” project focus of light pollution effects on insect populations.

In Fulda, the project is managed by the Environmental Center Fulda, which has an established network for acquiring citizen scientists and is widely known for environmental education, advice, and information. Fulda’s proximity to Rhön International Dark Sky Reserve gives the location the globally unique combination of dark sky city and dark sky reserve status, which both follow the philosophy of transforming people’s impressions of natural darkness at night as an ecosystem that needs protection. The ongoing public relations work for the relatively new certification as a “dark sky city” will be used to draw attention to the citizen science campaigns, and vice versa. People interested in environmental conservation are exposed to Fulda’s tasks and responsibility as an IDA dark sky city and learn how to contribute to limiting light pollution in their own homes and gardens. Both projects go hand-in-hand and profit from synergy. In the first year, a cooperation was initiated with the Fulda Canoe Club to activate and empty the emergence traps, as the boats could offer better access from the water site.

The start of the citizen scientist involvement and environmental education programs was significantly affected by the COVID-19 pandemic, which coincided with the planned start of the community involvement and the taxonomic workshops. COVID-19 regulations in Germany were such that in-person activities were severely restricted at some times and locations throughout the first year. Nevertheless, the citizen science activities started in the communities in small numbers and with the necessary precautions to limit the spread of potential infection. Citizen scientists visited the monthly insect sampling sites to help with the light traps and also to learn about environmentally friendly lighting in ongoing conversations during the pandemic-friendly outdoor activity. The first insect classification sessions were held in small groups with interns and volunteers. In Neuglobsow, workshops could be conducted due to the coordinators’ teaching exemption to give lessons to students during the lockdown. Workshops with three different school classes of 17 to 25 students in the age range of 10 to 18 were held at the community center in Neuglobsow and in the classroom.

## 4. Discussion

“Tatort Streetlight” creates public nightscapes for both human well-being and insect welfare, “welcoming insects back into our anthropogenic architecture to balance ecosystem services needed for the quality in life that we are used to”. This citation taken from the art project “Animal Estate” by Fritz Haeg (https://www.fritzhaeg.com/garden/initiatives/animalestates/main.html, accessed on 9 December 2021) demands a new perspective on insect decline, which is strongly bound to anthropogenic impact, and for too long has disregarded the needs of ecosystems and wildlife. Citizen scientists worldwide are needed to analyze these effects and create solutions, which consider humans as parts of ecosystems, who can create architecture that can benefit both human well-being and insect welfare. The first results in the project “Tatort Streetlight” show the complex character of the endeavor to bring a new insect-friendly road lighting design into praxis and the potential pitfalls for interdisciplinary communication.

### 4.1. Insect Monitoring

The preliminary results of the first project year that are shown here indicate that road lighting near aquatic ecosystems presents a long-ignored threat to biodiversity, particularly for highly threatened aquatic taxa. Nocturnal insects of diverse orders, including protected species, are fatally attracted to light sources [39]. For example, the orders of mayflies and caddisflies (Ephemeroptera and Trichoptera) are listed with 51% and 43% of endangered species according to the IUCN library (https://portals.iucn.org/library/taxonomy/term/44249, accessed on 9 December 2021). Members of these aquatic insect groups often emerge collectively at single events in which billions of individuals, equivalent to tons of biomass, leave the water body into the airspace over several hours. For instance, the emergence flights of the burrower mayflies (Ephemeroptera; Hexagenia) along the Upper Mississippi River and the Western Lake Erie Basin can be observed by radar, where a decline in emergence flights by over 50% was recorded in a recent study [64]. A mayfly, stonefly, and caddisfly specialist group was established by the IUCN to promote the conservation of these species and their habitats around the world (https://www.iucn.org/commissions/ssc-groups/invertebrates/mayfly-stonefly-and-caddisfly, accessed on 9 December 2021). In “Tatort Streetlight” data, the orders Ephemeroptera and Trichoptera constitute a significant proportion of the insects collected. The differences in numbers of individuals caught at day and night-time indicate a high vulnerability of these insect groups to road lights close to freshwater bodies, which corroborates prior studies [39,65]. Another group that might be disproportionally affected by artificial light near freshwater ecosystems contains aquatic Heteroptera [66], which were also found significantly more often in the night-time samples. Detailed analyses that would be needed to investigate the vulnerability of aquatic taxa within this order were beyond the scope of the present study but are planned to be included in future studies. The differences in the sampled numbers between the different communities can be explained by the distance of the eclector traps to the next water body, as Ephemeroptera and Trichoptera species show highly variable dispersal behavior [67]. Secondly, the water quality might be more favorable in the lakes at Krakow am See and Neuglobsow than in the urban river in Fulda, where the lowest numbers of aquatic insects were recorded (Figure 6). Our data also indicate a rather wide dispersal from the water body, because even in Gülpe high numbers of Trichoptera were attracted to the luminaires, although they are located at over 450 m distance to the water body. In Krakow am See, the eclector traps are installed directly adjacent to the waterbody and high numbers of Ephemeroptera were recorded, particularly in June and August 2020 (Figure 6). Secondly, the variations can be explained by the differences in duration of illumination through the night. For example, in Fulda all lights were turned off after 22:30, and although the eclector traps are close to the water body, very low numbers were caught. The same applies to the group of Lepidoptera. Although mainly nocturnal Lepidoptera were caught, the day and night comparison did not present significant differences, probably due to insufficient total numbers. Given the partial turning off of some of the lights, this result could be an indication of the higher activity of nocturnal moths at later periods during the night. The preliminary data, however, are insufficient to prove such effects. Further research on the occurrence of insect orders throughout the night is urgently needed. What our preliminary data do show is that road luminaires are attracting protected and common insect orders in high numbers. There is an urgent need to reduce the adverse effects of road lighting insects, although existing environmental protection regulations are insufficient tools in regards to outdoor lighting systems [68,69].

Citizen scientists can play a critical role in achieving the aims of regulations [70]. Global monitoring data relating to insect attraction to light sources are crucial to understand the impacts on insect populations and to make the case for greater protection against the impacts of ALAN. Citizen science can provide human resources to (a) monitor the occurrence of the insects, (b) measure the night sky brightness, and (c) increase awareness of the adverse effects of illumination for better acceptance of necessary regulations. Technological solutions and regulations to mitigate the adverse effects of outdoor lighting can be inexpensive and easy to obtain [71].

### 4.2. The Night Sky Brightness

Some of the areas in the partner communities of the “Tatort Streetlight” project provide night sky brightness conditions that are rare in Europe, meaning protection of the quality of the night sky is highly important. To avoid rebound effects of private lighting, it is important to actively engage in public debate over lighting at night with evidence-based arguments. As light is associated with positive feelings, the development to less bright outdoor illumination needs to be carefully introduced and an assessment of the stakeholder opinions regarding the new road lighting design and the results of insect monitoring are rather important. Today, the data on night sky brightness in the project areas are scarce. “Tatort Streetlight” will initiate further measurements at locations close to the areas where road lighting will be changed. A recent study showed that only 13% of night-time radiance could be traced back to the road illumination in Tuscon, Arizona, US [72]. This was measured by the differences in satellite images taken at various levels of the road lighting, which was intentionally dimmed for this purpose. However, the sensitivity spectrum of the VIIRS DNB satellite sensor ranges from 500 to 900 nm only. This spectrum is insufficient to detect the short wavelengths emitted by many LED road lighting systems and thus further measurements from the ground are generally needed to identify altered lighting conditions [73]. It is important to highlight that the data obtained in Tucson cannot be generalized to all scenarios of light pollution regarding road lighting. Firstly, Tucson has had an optimized dark-sky-friendly road lighting system installed. Secondly, the impact of road illumination is presumably much higher in rural areas with less business lighting from shops, offices, and advertisement billboards. Private lighting from houses and gardens might have an equally high impact as the public lighting in such areas. Thirdly, different effects such as air pollution can disturb satellite night-time radiance measurements, making ground-based measurements very important, as shown during the COVID-19 lockdown near Berlin [53].

### 4.3. The Citizen Science Approach and Stakeholder Analysis

At the intersection of public lighting and safety, scientific biodiversity research, and governance, this project offers many entry points for the public. It offers communication, environmental education, and active participation at different levels up to active co-research by laypeople and hobby entomologists in the field of citizen science. However, the citizen science activities of “Tatort Streetlight” had just started when the lockdown to prevent COVID-19 began in March 2020. Projects with established citizen science infrastructure and engaged participants could benefit from increased citizen science data numbers and quality within the pandemic lockdown situation [74,75]. The first results from “Tatort Streetlight” indicate stakeholder interest in the subject, although the citizen science motivation and participation in “Tatort Streetlight” were clearly hampered by the COVID-19 outbreak due to the lack of established communities and digital infrastructure. A useful precondition was the employment of one of the coordinators as teacher, who was able to continue teaching students during the pandemic. Thus, a focus on citizen involvement in educational activities was followed.

The approach of offering environmental education via preschool and school children is beneficial to the project in two ways—it can initiate interest about entomology at an early stage and it opens up citizen science to diverse groups. Entomological taxonomy has been declining in the past decades, resulting in a range of poor taxonomic identifications of insects or unrepeatable studies [76]. The history of entomology comprises different areas, with rising numbers of entomologists until the last century, mainly from Europe [77]. However, the numbers are now declining and the demographics of entomologists lack inclusiveness. The field of entomology used to be purely male-dominated [60]. The first women that were active in the field were only recorded at the beginning of the 20th century [78]. A study about citizen science participation in environmental projects in the UK revealed that men were more likely to participate than women. Furthermore, people identifying as being from white ethnic groups were more likely to participate than those identifying as being from minority ethnic groups, and participation by women from minority ethnic groups was particularly low [79]. Aside from the ethical importance of inclusiveness, the scientific process benefits from the heterogeneity of perspectives that various ethnical demographics can provide, widening the scope of environmental science in terms of perceptions and interactions between humans and insects [80]. Both articles discuss education as a critical entry point to environmental or entomological studies, stating that an investment in training of future entomological scientists is needed. “Tatort Streetlight” aims to open up the subject of entomology to divers groups and provides education to school children as well as to all interested stakeholder groups.

Furthermore, it is important to stay in contact with decision makers and community authorities and to emphasize the threat to biodiversity. Each community can help to achieve UN Sustainable Development Goals (https://sdgs.un.org/goals, accessed on 9 December 2021) and the EU 2030 Climate Target Plan (https://ec.europa.eu/clima/policies/eu-climate-action/2030_ctp_en, accessed on 9 December 2021) by regulating public lighting to meet these needs. It is intended that the partner communities could act as role models to other communities in promoting regionally protected nightscapes. The proposed measurements for night sky brightness will be evaluated and could provide levels for standards in the future. Such a standardized method will be an essential factor for protecting biodiversity and saving energy [54]. The technical solution for insect protection through the insect-friendly road lighting design might become mandatory for the modernization of road lighting according to the third draft amending the Federal Nature Conservation Act (https://www.bmu.de/fileadmin/Daten_BMU/Download_PDF/Gesetze/3_aenderung_bnatschg_bf.pdf, accessed on 9 December 2021). The involvement of all relevant stakeholders is of great importance in order to consider all aspects of the benefits and possible adverse effects as comprehensively as possible.

However, in the eyes of a lighting engineer, the proposed design could be judged as an inefficient technology, as the light distribution presents a steep angle and does not illuminate efficiently between the road luminaires. It is, thus, an important future task to communicate the importance of reducing the light emissions into insect and wildlife habitats by shielding the luminaires. Further developments of the optics, to produce the most efficient light distribution with shielded lamps must become a new aim in luminaire development.

The new roadway luminaire and lighting design developed in the project will set the standard for future outdoor lighting and make a major contribution to species conservation. As with Haeg’s “Animal Estates”, we aim to broaden the pool of stakeholders in road lighting to welcome insects back into our living environment. “Animal estates” invite wildlife back into anthropogenic life, intending to eradicate the strict, arbitrary, and obsolete boundaries that humans have established between the anthropogenic lifestyle and wildlife habitats. This approach should prevail in order to promote biodiversity across the board.

## 5. Conclusions

A detailed understanding of the anthropogenic impacts, such as outdoor lighting, on insects is timely and urgent to safeguard biodiversity. Citizen science is a vital tool to monitor both insect occurrence and the impacts of ALAN on the environment. Furthermore, citizen science can increase public awareness and assist in developing solutions. Developed with societal feedback, the results of such activities are often better accepted by the community [70].

Environmental education is a critical factor in increasing awareness and citizen science engagement. In particular, education around entomology is a helpful tool to increase public perception of the “critters” that provide important ecosystem services and require urgent protection from anthropogenic stressors [81].

Current environmental protection legislation lacks regulations for the protection of species and habitats from ALAN, especially those considered “normal”, i.e., those without any special protection status, but which are equally important as food sources in the trophic system [68,69]. “Tatort Streetlight” is a citizen science project with the potential to establish and support regulations to reduce the adverse effects from road lighting on biodiversity and the night sky and to offer insect-friendly lighting solutions.

The ecological functions insects provide are needed everywhere, not only in protected areas. The development of this unique “Tatort Streetlight” luminaire represents an advancement in terms of bringing the protection of biodiversity into everyday landscapes. While this project focuses on the protection of insects, the approach presented here is adaptable to other lighting solutions to reduce the impact of ALAN on biodiversity [82].

## Figures and Tables

**Table 1:** Necessary requirements according to EN 13201 to ensure traffic safety. P-classes 3 and 4 shown with corresponding illuminance requirements [36].

Lighting Class	*Ē*_av_ (lx)	*E*_min_ (lx)	*E*_av,max_ (lx)	TI Maximum %
P3	7.50	1.50	11.25	25
P4	5.00	1.00	7.50	30
P5	3.00	0.60	4.50	30

**Table 2:** Measured geometries of the project areas. Illustrations by Pérez-Vega.

Project Area	Street Width	Pole			Luminaire Type
		Height (h)	Distance (d)	Ratio d/h	
Krakow am See	2.7 m	3.44 m	25 m	7.3	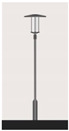
Neuglobsow	4.0 m	3.30 m	30 m	9.1	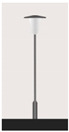
Gülpe	5.4 m	4.40 m	30 m	4.8	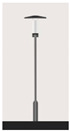
Fulda	2.8 m	4.33 m	50 m	11.6	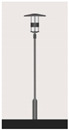

## Data Availability

The data presented in this study are available on request from the corresponding author. The data are not publicly available due to the preliminary status. The meta-data of the different study areas are not comparable and thus the analysis could lead to wrong conclusions. After the results of the project “Species Protection through Environmentally Friendly Lighting” have been published open access of the data will be provided.

## Supplementary Materials

The following are available online at https://www.mdpi.com/article/10.3390/insects12121117/s1: Figure S1: Overview of the project regions in Germany. Figure S2: Locations of insect traps in the project community in Krakow am See (Mecklenburg-Vorpommern). Figure S3: Locations of insect traps in the project community in Neuglobsow (Brandenburg). Figure S4: Locations of insect traps in the project community in Gülpe (Brandenburg) and on the river Havel. Figure S5: Locations of insect traps in the project community in Fulda (Hessen). Figure S6: Relative numbers of flying insects trapped per hour in all partner communities. Figure S7: Relative numbers of flying insects trapped per hour in all partner communities. Figure S8: Detailed distribution of insects caught per trap hour in the four partner communities separated for day versus night.
