# Supplementary material for: Towards Insect-Friendly Road Lighting—A Transdisciplinary Multi-Stakeholder Approach Involving Citizen Scientists
Source: Insects. 2021 Dec 14;12(12):1117. doi: 10.3390/insects12121117 (PMC8706979; doi:10.3390/insects12121117)
Supplement: Supplementary file 1 [file insects-12-01117-s001.zip › insects-1484436-proofed-s.pdf]

insects

Supplementary Information

Towards insect-friendly road lighting – a transdisciplinary multi-stakeholder approach involving citizen scientists

Sibylle Schroer [1*], Kat Austen [1], Nicola Moczek[2&3], Gregor Kalinkat [1], Andreas Jechow [1], Stefan Heller [1], Johanna Reinhard [1], Sophia Dehn [4], Charis I. Wuthenow [5], Martin Post-Stapelfeldt [6] , Roy H.A. van Grunsven [7], Catherine Pérez Vega[1&8], Heike Schumacher [9], Leena Kaanaa [9], Birte Saathoff [9], Stephan Völker [9] and Franz Hölker [1]

[1] Leibniz Institute of Freshwater Ecology and Inland Fisheries, 12587 Berlin, Germany; kat.austen@igb-berlin.de; gregor.kalinkat@igb-berlin.de;andreas.jechow@igb-berlin.de; stefan.heller@igb-berlin.de; johanna.reinhard@igb-berlin.de; catherine.perez@igb-berlin.de; franz.hoelker@igb-berlin.de

[2] PSY: PLAN Institute for Architectural and Environmental Psychology, 10245 Berlin, Germany; moczek@psyplan.de

[3] Museum für Naturkunde Berlin, Leibniz Institute for Evolution and Biodiversity Science, 10115 Berlin, Germany

[4] NABU RVWesthavelland e.V., 14715 Milower Land, Germany; aube@nabu-westhavelland.de

[5] Umweltzentrum Fulda-Zentrum für Nachhaltigkeit, Gartenkultur und Tierpädagogik e.V., 36041 Fulda, Germany; Charis.Wuthenow@fulda.de

[6] Naturpark Nossentiner/Schwinzer Heide e.V., 19395 Plau am See, Germany; martinpost1986@gmail.com

[7] Dutch Butterfly Conservation, PO-box 506 6700 AM Wageningen, The Netherlands; roy.vangrunsven@vlinderstichting.nl

[8] Department of Biology, Chemistry, and Pharmacy, Institute of Biology, Freie Universität Berlin, 14195 Berlin, Germany

[9] Chair of Lighting Technology TU Berlin, 10587 Berlin, Germany; heike.schumacher@tu-berlin.de (H.S.); leena.kaanaa@tu-berlin.de; birte.saathoff@tu-berlin.de; stephan.voelker@tu-berlin.de

* Correspondence: sibylle.schroer@igb-berlin.de; Tel.: +49-306-4181-717

1. Maps of the communities and the exact trap settings

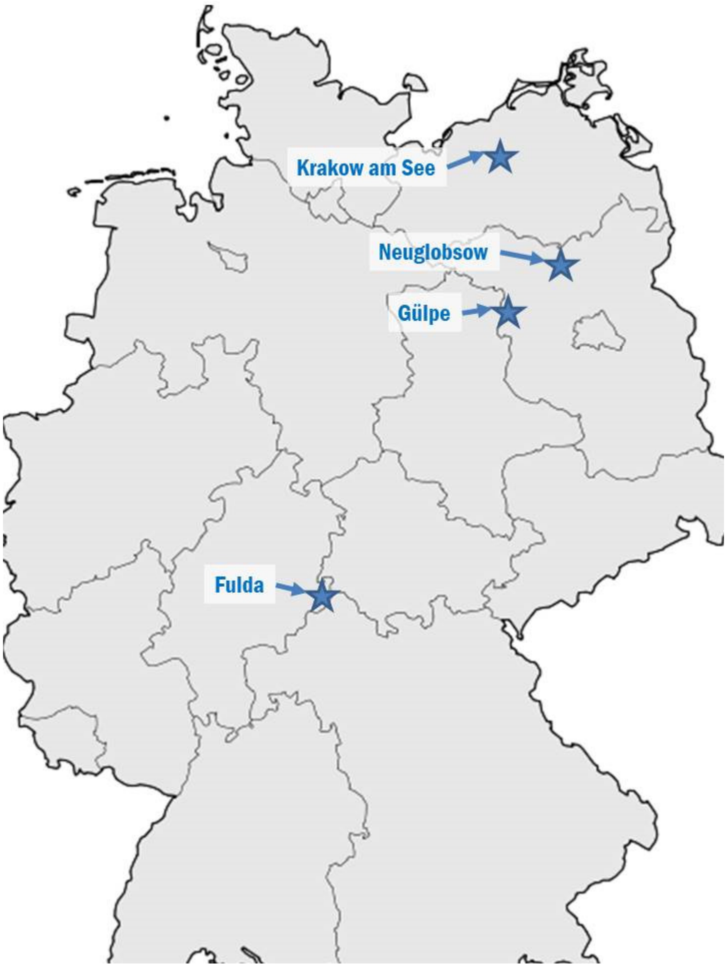

Figure S1: Overview of the project regions in Germany implemented in open street map (https://www.openstreetmap.org).

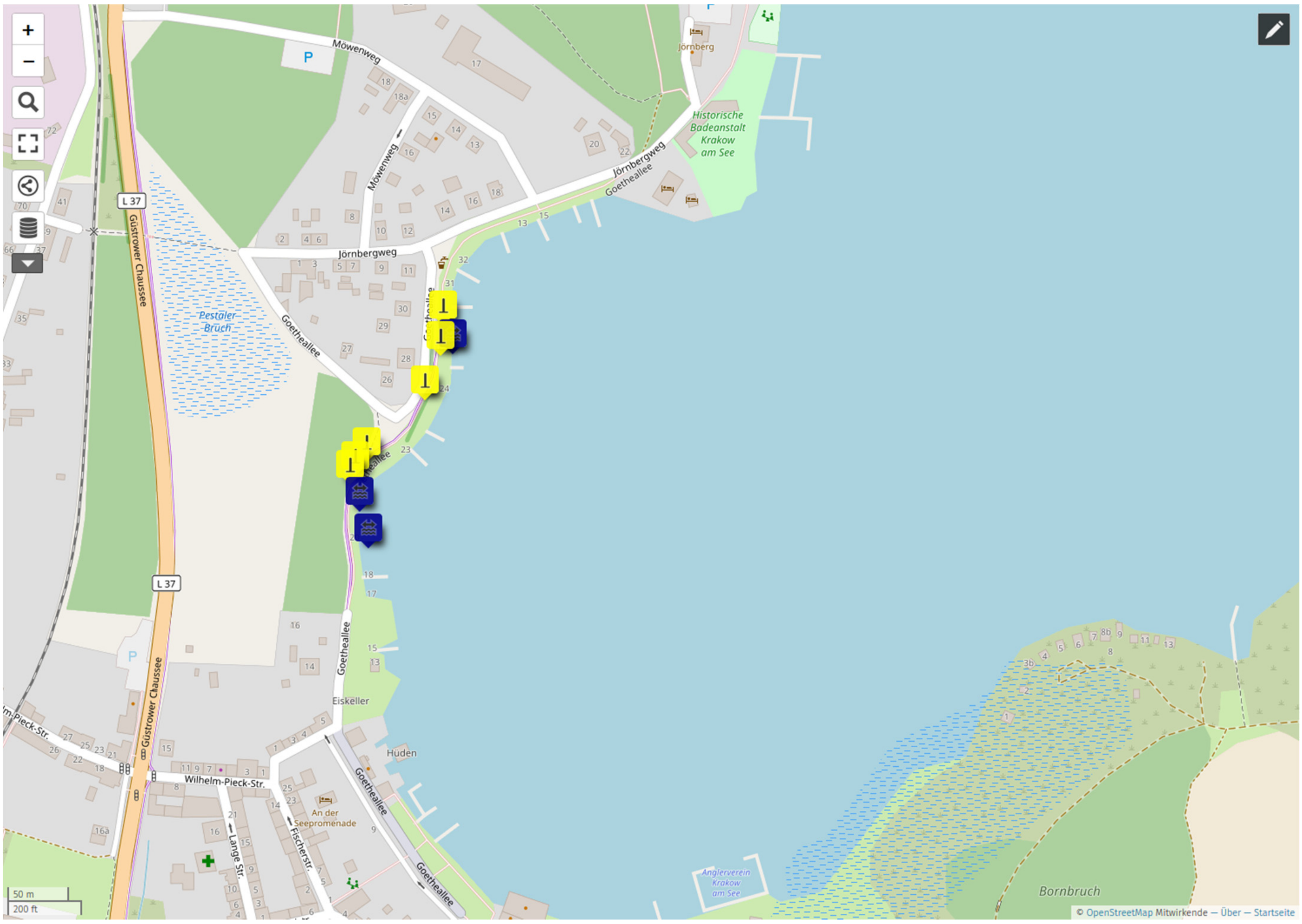

Figure S2: Locations of insect traps in the project community Krakow am See (Mecklenburg-Vorpommern). Black pole on yellow ground symbolizes air eclector traps mounted at the road lights. The distance between the air eclector traps is according to the road lighting poles. Black waves on blue ground symbolize floating aquatic emergence traps at a nearby waterbody), which were positioned at the closest point in the freshwater body with manageable access.

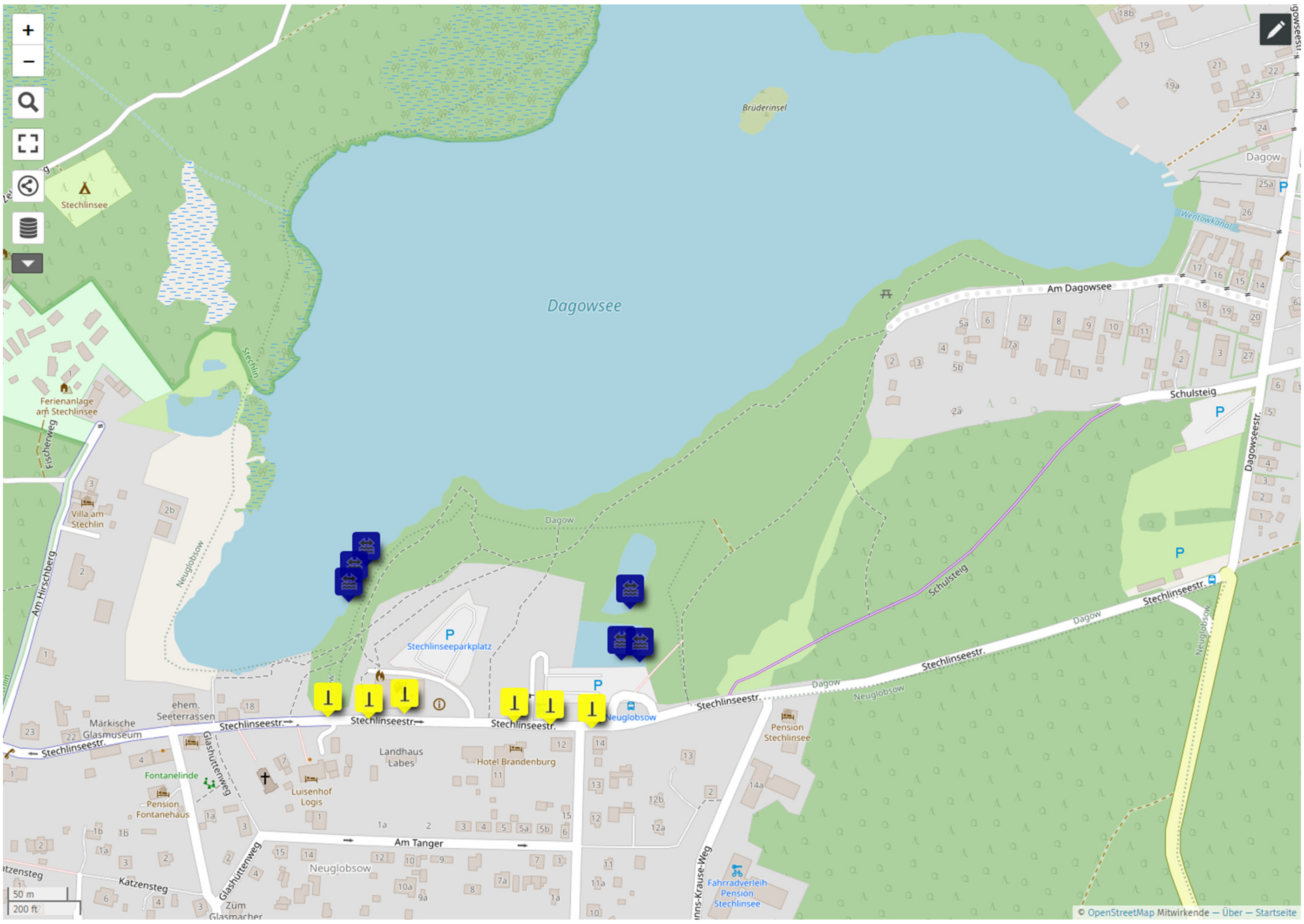

Figure S3: Locations of insect traps in the project community Neuglobsow (Brandenburg). In this location there are additional emergence traps installed to cover multiple water bodies (one major natural lake, Dagowsee, and two artificial ponds).

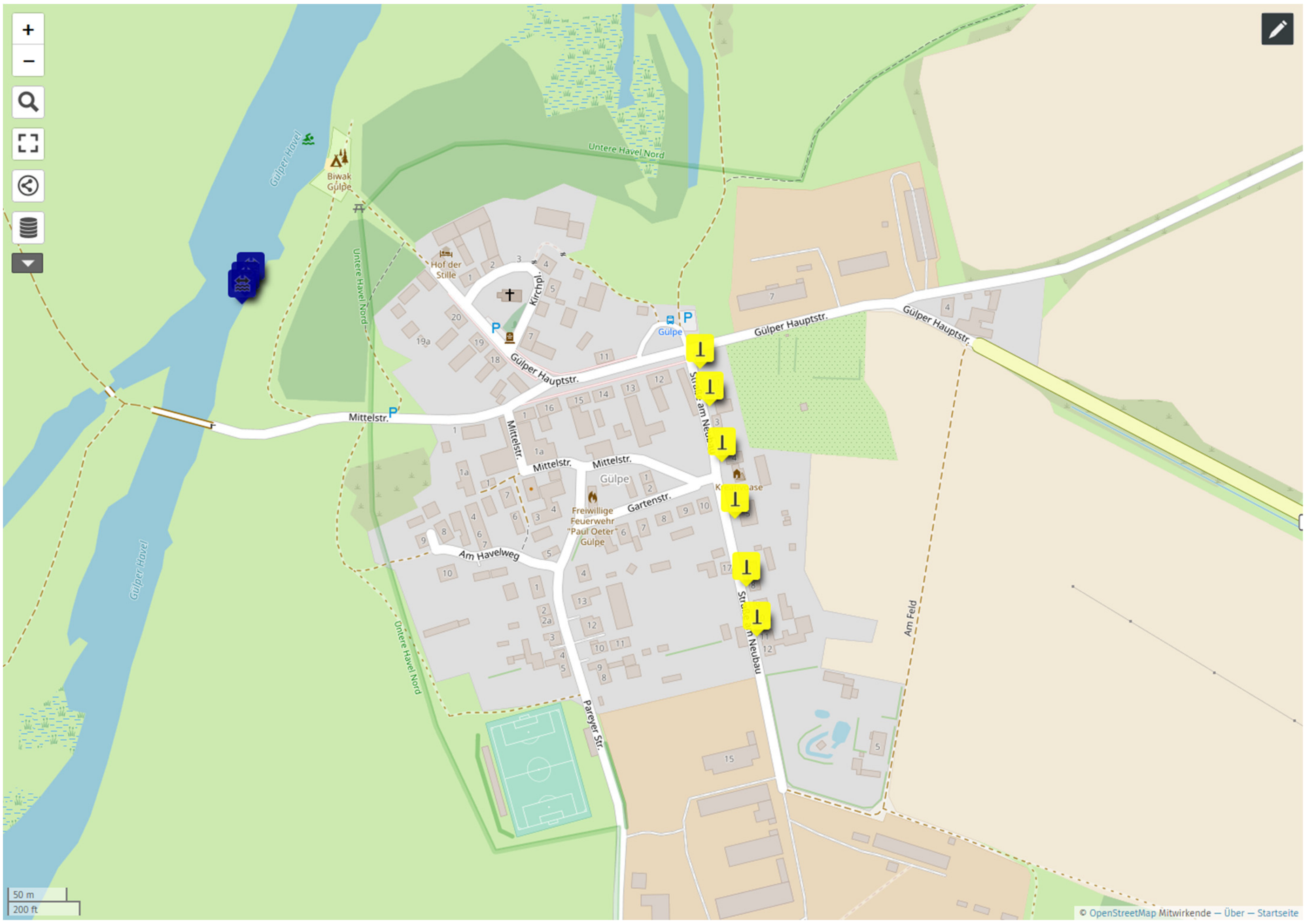

Figure S4: Locations of insect traps in the project community Gülpe (Brandenburg) and on the river Havel.

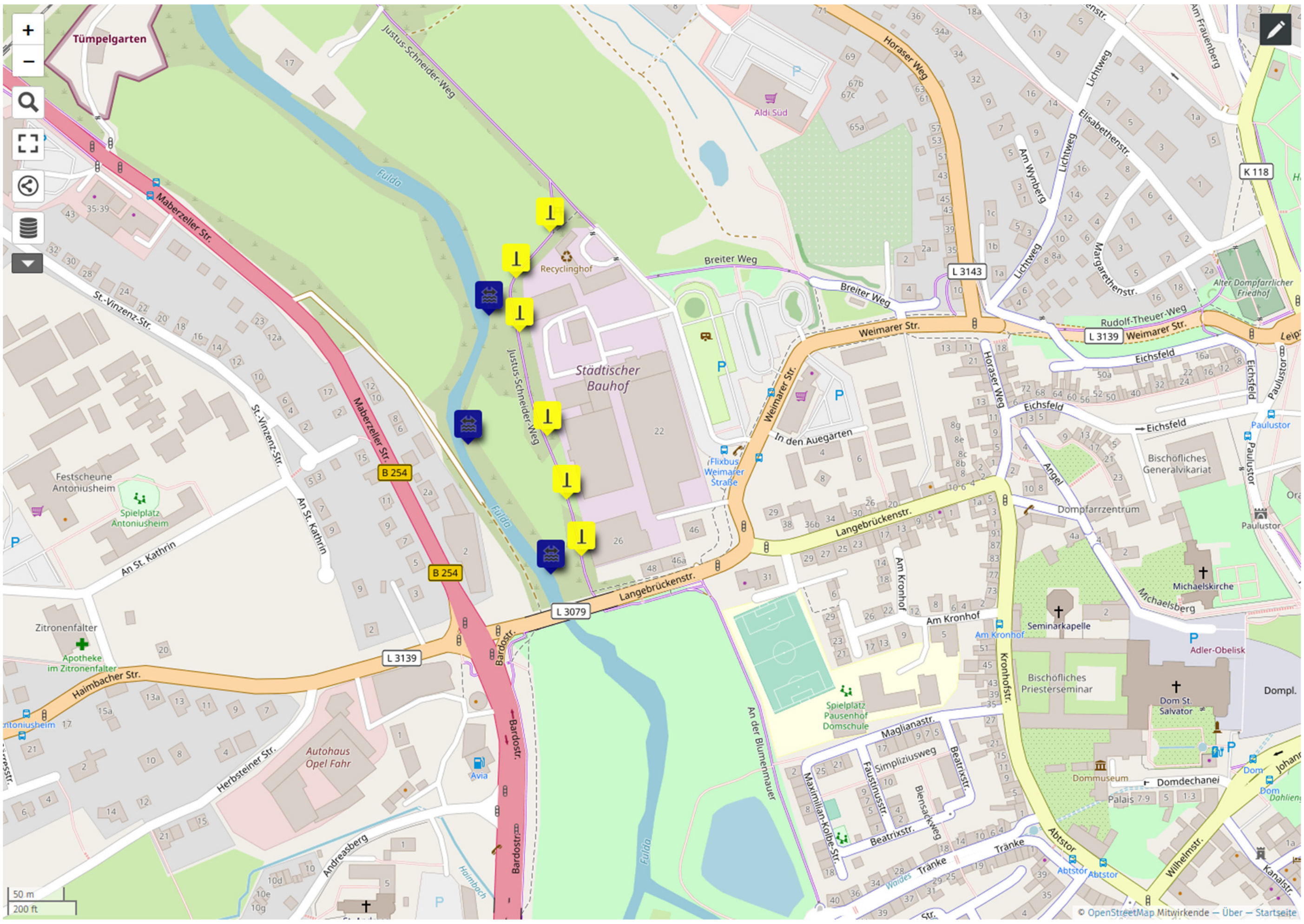

Figure S5: Locations of insect traps in the project community Fulda (Hessen).

2. Insect monitoring results

Figure S6

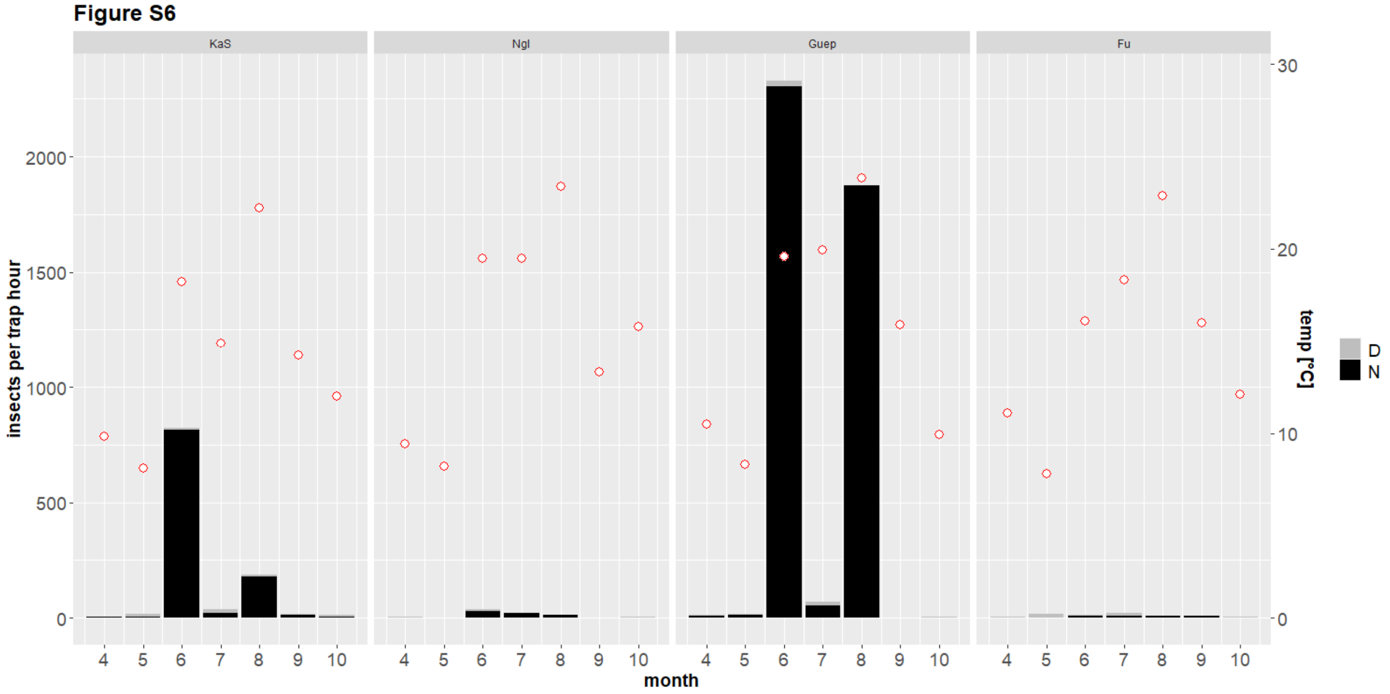

Figure S6: Sums of relative numbers of flying insects trapped per hour in all partner communities (bars) during day (grey) and night (black). Mean 24 hour temperature of the sampling dates is also shown (red circles). The most extreme values for insect numbers are driven by Nematocera in Gülpe and mass emergence events of Ephemeroptera in Krakow am See. (KaS = Krakow am See, Ngl = Neuglobsow, Guep = Gülpe, Fu = Fulda)

Figure S7

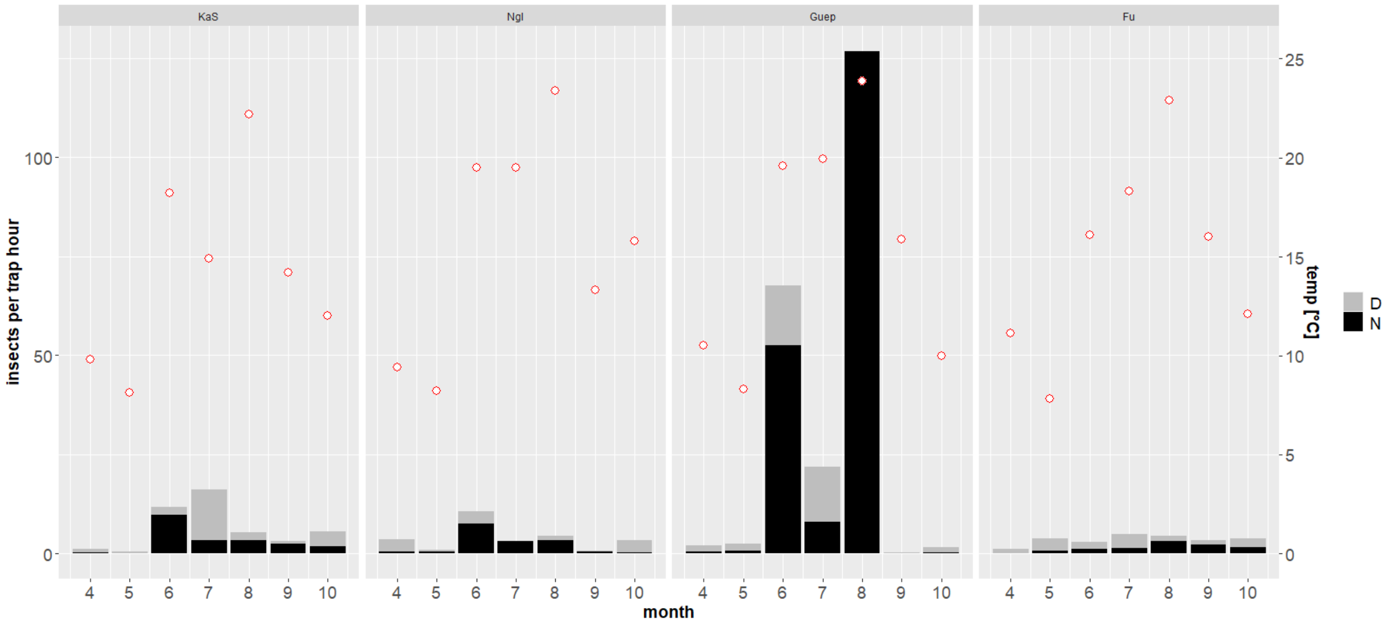

Figure S7: Sums of relative numbers of flying insects trapped per hour in all partner communities (bars) during day (grey) and night (black). Mean 24 hour temperature of the sampling dates is also shown (red circles). For this plot Nematocera and Ephemeroptera were skipped to improve the visibility of the other taxa. (KaS = Krakow am See, Ngl = Neuglobsow, Guep = Gülpe, Fu = Fulda)

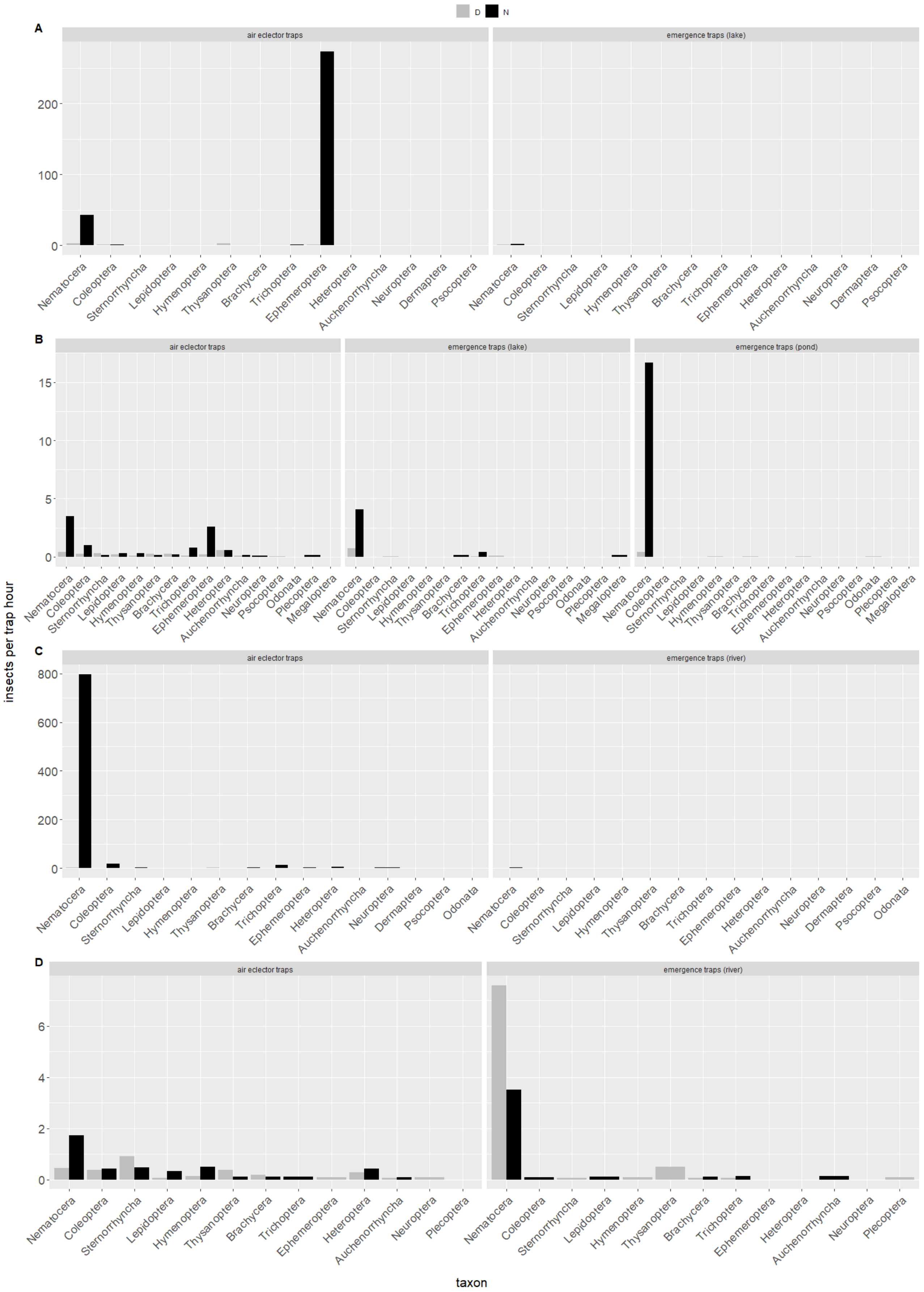
D
N
A
air eclector traps
emergence traps (lake)
B
air eclector traps
emergence traps (lake)
emergence traps (pond)
C
air eclector traps
emergence traps (river)
D
air eclector traps
emergence traps (river)
insects per trap hour
taxon
Nematocera
Coleoptera
Sternorrhyncha
Lepidoptera
Hymenoptera
Thysanoptera
Brachycera
Trichoptera
Ephemeroptera
Heteroptera
Auchenorrhyncha
Neuroptera
Dermaptera
Psocoptera
Odonata
Plecoptera
Megaloptera

Figure S8: Detailed distribution of insects caught per trap hour in the four partner communities separated for day (grey) versus night (black). Daytime samples were collected from dawn until dusk and nighttime samples from dusk till dawn. Results of the different trap types (air eclector and emergence are presented forA= Krakow am See, B = Neuglobsow, C = Gülpe, D = Fulda. In Neuglobsow (B) two different freshwater ecosystems are sampled with emergence traps (see Figure S3).
